# Surface roughness and cyclic fatigue resistance of a novel shaping system: An *in-vitro* study

**DOI:** 10.1371/journal.pone.0302551

**Published:** 2024-05-02

**Authors:** Beliz Özel, Güher Barut, Elif Delve Baser Can

**Affiliations:** 1 Endodontics, Academic Center for Dentistry, Amsterdam, Netherlands; 2 Endodontics, Yeditepe University Faculty of Dentistry, Istanbul, Türkiye; Universidade Federal Fluminense, BRAZIL

## Abstract

Recently developed Nickel-Titanium (NiTi) instruments with practical changes have resulted in safer instrumentation. In addition, topographical features on the file surface are a contributing factor to clinical durability. Therefore, this study aimed to investigate both the cyclic fatigue resistance and the roughness change of MTwo and Rotate instruments (VDW, Munich, Germany). Each instrument (n = 6/each group) was scanned with an atomic force microscopy prior to and after instrumentation. In addition, cyclic fatigue testing was conducted for each instrument (n = 11/each group) with stainless-steel blocks, including 45°-60°-90° degrees of curvature milled to the instruments’ size. The roughness parameters increased for both systems after instrumentation (p<0.05). Both systems presented an increased roughness following instrumentation (p<0.05). The cyclic fatigue resistance was lowest at 90° for both systems (p<0.05), whereas the Rotate files presented a higher resistance than that of the Mtwo files (p<0.05). Compared to the Mtwo files, Rotate files presented better resistance, while the resistance decreased as the curvature increased.

## Introduction

Over the last three decades, conventional stainless-steel files have been replaced by nickel-titanium (NiTi) instruments and have been widely used due to their unique properties, such as shape memory, superelasticity, and resistance to torsional fracture [1]. However, despite the many advantages of NiTi instruments, unexpected fractures may occur during root canal preparation, even in the absence of any previously visible deformations on the instrument’s surface [2, 3].

An overload resulting from cyclic or torsional stresses that exceed the instrument’s strength will eventually lead to fatigue and further separation [3]. Cyclic fatigue occurs when repeated cycles of tension and compression lead to structural breakdown and eventual fracture of an instrument that has been bent beyond its limit in curved canals [4].

Previous reports have shown irregularities characterized by milling marks, multiple cracks, and pits on the surfaces of NiTi instruments that can lead to the progression of cracks at the material surface with repeated use [5]. Surface treatments, such as electropolishing, electrical discharge machining, or physical vapor deposition, have been introduced to improve surface smoothness and delay crack or wear progression, thus increasing the fatigue resistance of the files [6–8].

In addition to surface treatments, manufacturers have developed new designs and heat-treated NiTi alloys to increase the efficiency of instrumentation [9]. Heat-treated NiTi instruments, which undergone a proprietary heating and cooling processes after machining, have been reported to exhibit an increased flexibility, higher cyclic fatigue resistance and better canal centering ability [10, 11]. One of these is a “blue treatment” in which a visible titanium oxide layer is responsible for the distinctive blue colour that remains on the surface as a result of the process, which causes the instrument exhibit an improved fatigue resistance [11–13].

Several methods have previously been used to evaluate the effectiveness of surface treatments. Scanning electron microscopy (SEM), which has long been a standard in evaluating surface changes, uses an electron beam operated under a vacuum to produce a two-dimensional “photographic” image of the samples; however, SEM cannot directly provide reliable quantitative data [6]. Atomic force microscopy (AFM) provides the highest resolution atomic-scale characterization of material surfaces at the nanometer scale and requires little or no sample preparation [14]. While SEM only provides a 2D image, AFM offers a high-resolution 3D profile on the nanoscale by measuring the forces between a flexible probe and surface at extremely short distances of 0.2–10 nm. This interaction is recorded by calculating the small force between the probe and the surface [15].

Over the years, surface characteristics and fatigue resistance have caught the attention of researchers. Yamazaki-Arasaki reported increased wear following root canal shaping with NiTi files in resin blocks [16]. Likewise, Can Saglam et al. reported increased surface damage on retreatment files following the use [17]. One recent study by Ertugrul et al. [18] reported a higher cyclic fatigue life of Rotate compared to MTwo with a single curvature of 60° and proposed that surface topography is important and may affect the cyclic fatigue resistance. Additionally, it has been claimed that the fatigue life of endodontic files may be shortened with an increase in surface roughness [19, 20]. Thus, it is important to perform a topographical analysis of the instrument to explain its influence on the instrument’s resistance [16].

MTwo rotary system (VDW, Munich, Germany) was produced using the conventional NiTi alloy with an “S-shaped” cross-sectional design and a non-cutting tip, less canal transportation after root canal preparations of curved canals, whereas Rotate (VDW, Munich, Germany) provided an improved version of MTwo system with an adapted S-shaped cross section, reduced file sequence and an increased instrument flexibility following the use of blue-treated NiTi alloy [21, 22].

Many studies have compared the cyclic fatigue resistance of MTwo and Rotate instruments with different cross-sectioned and manufactured NiTi rotary instruments [23–25], however there has yet to be a study published to date on the surface roughness of Mtwo and Rotate instruments before and after instrumentation. Thus, to determine whether surface irregularities occurred throughout usage, this study aimed to compare the cyclic fatigue resistance of Rotate and MTwo with the angles of 45°-60°-90° of curvature and evaluate changes in the surface topographies after using both instruments. The null hypothesis was that the surface roughness and cyclic fatigue resistance within and between tested groups exhibit no difference.

## Materials and methods

### Sample size calculation

The sample size required for AFM scanning was calculated following the effect size calculation of the results of a previous study [26] with an effect size of d = 1.88, and an estimation of 12 samples (n = 6) is required to detect a difference between groups with an a of 0.05 and a 1-b of 0.80.

The sample size required for cyclic fatigue testing was calculated following the effect size calculation of the results of a previous study [27] with an effect size of d = 0.47, and an estimation of 22 samples (n = 11) is needed to detect a difference between each group with an a of 0.05 and a 1-b of 0.80.

### Selection of the teeth

Written consent was obtained from patients after approval by the Research Ethics Committee of Yeditepe University (approval number 1767) to use their extracted teeth in the study. Twelve human upper first molar teeth with a minimal root length of 16 mm, extracted for orthodontic or periodontal reasons between 11/08/2023 and 11/10/2023, were selected and then stored in 0.5% chloramine T solution at 4°C for up to 2 months following extraction. The selected teeth were then randomly divided into two main groups (n = 6) according to a randomized sequence generator (random.org).

### AFM scanning

Prior to the preoperative scanning, MTwo #20.06 and Rotate #20.05 (n = 6) were collected, and the shaft portion of each file was marked as a reference point to help position the sample on the scanning stub. Three random points at 5 mm from the tip were selected, and their coordinates were recorded for use in secondary scans. Areas of 5x5 μm^2^ of each point were scanned under the ‘intermittent’ mode using a cantilever probe at a force constant of 40 nN/nm. The mean roughness average (Ra) and root mean square (RMS) values (nm) were calculated with the XEI image processing tool (version 1.7.6) [16]. Ra, the roughness average, is the arithmetic mean of the absolute surface profile values and it is the most commonly used parameter for measuring the roughness of samples. However, due to recording the ‘absolute’ values, Ra was inadequate in distinguishing peaks and valleys. To fully characterize such features, a detailed parameter was added. RMS (Rq), root mean square, takes the mean of squared absolute values, is more sensitive to peaks and valleys on the sample surface, and provides detailed information about the test surface. The measurements were conducted in ‘intermittent mode’, in which the cantilever of the AFM probe taps the surface during scanning. This mode is usually chosen for samples with soft structures on their surfaces, such as polymers or thin films [28].

### Root canal instrumentation

Apical patency was gained with a #10 K-File (MAccess, Dentsply Maillefer, USA). Each file was used to shape the three canals of one human upper first molar tooth in total. Files reached the apical portion in three strokes. Canals were irrigated with 2 mL 5,25% sodium hypochlorite (NaOCl) between each file. The debris was cleaned from the instrument with gauze moistened with 5.25% NaOCl. The procedure continued the same for all tested files in each group (n = 6). All procedures were performed by the same operator, who had experience in rotary instrumentation systems.

Subsequently, files were immersed in an ultrasonic bath filled with distilled water for 180 seconds, followed by autoclave sterilization. Next, the same six files in each group were scanned with atomic force microscopy, using the initial coordinates. Mean Ra and RMS values were recalculated.

### Cyclic fatigue testing

**Fig 1:**
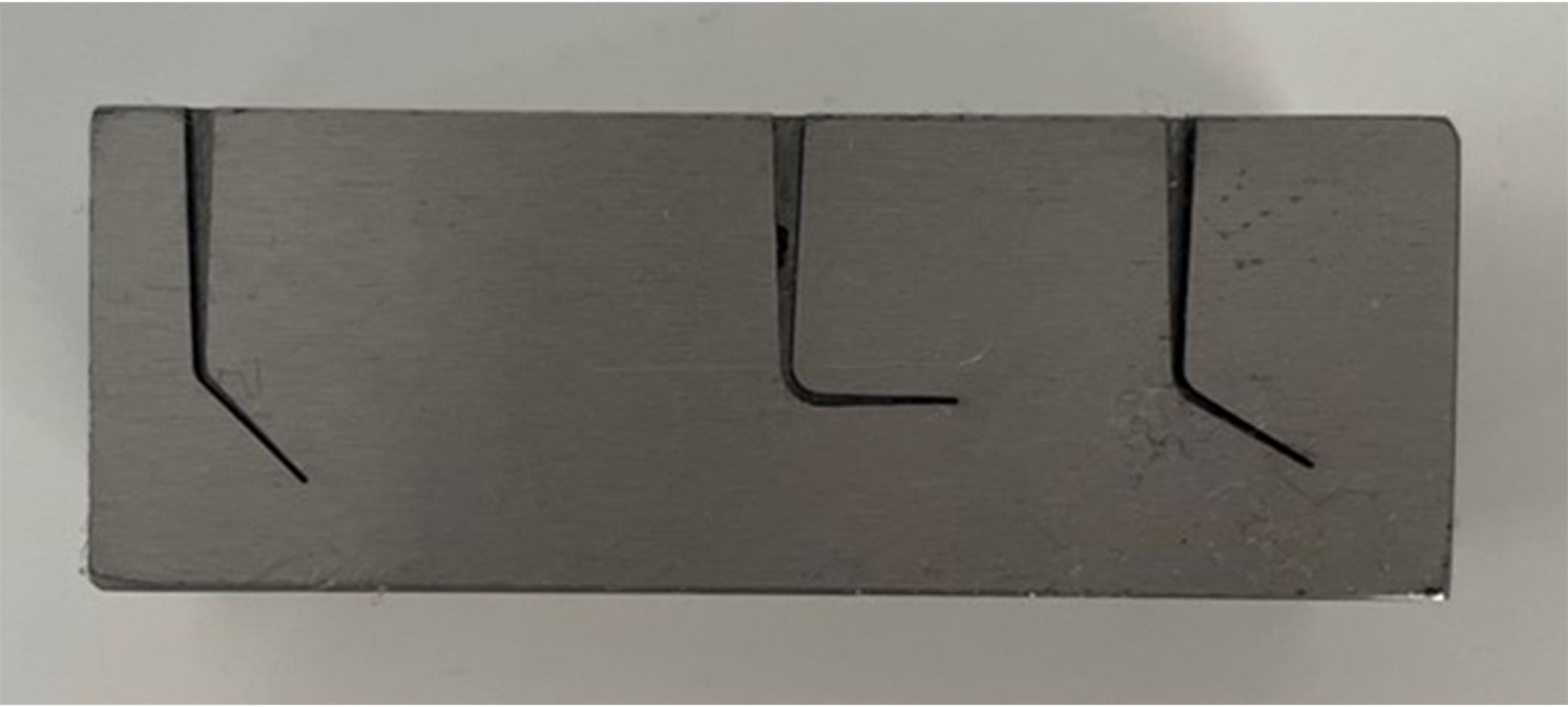
Cyclic fatigue test block.

Computerized artificial canals were designed specific to the instruments’ size and taper to provide a suitable trajectory during testing using CAD/CAM software (TopSolid, Evry, France) [27]. Each pattern of simulated canals was constructed according to the following parameters: (a) the size of each artificial canal was particular to the shaping system used and enlarged by 0.1mm to allow free rotation while generating minimal torque values, and (b) the curvature of each canal was determined following the method proposed by Pruett et al. [29] in which the radius and angle of curvature determine the severity of the curvature. Three simulated canals, 16mm in length, each with a 5-mm radius and angles of 45°-60°-90° of curvature, were designed as follows: (c) the center of curvature was pointed at 5 mm from the apical part of the canal, and (d) the depth of the artificial canals was machined to the maximum diameter of the instrument +0.2mm to allow the instrument to rotate freely inside. These patterns were machined onto a stainless steel block using a wire-cutting spark-erosion milling process (AgieCharmilles, GF, Switzerland) specific for each instrument (Fig 1).

The stainless-steel blocks were covered with tempered glass to prevent the instruments from slipping out and to allow observation of the fracture during testing. One drop of glycol was dripped inside the canals prior to testing to ensure lubrication. The static cyclic fatigue resistance test was performed with MTwo files size #20/.06 (n = 11) and Rotate files size #20/.05 (n = 11) different from AFM scanning, rotated with a torque-controlled electric motor (VDW, Munich, Germany) at 300 rpm and 2.5 N/cm torque at room temperature. The experimental procedure was performed by a single, experienced operator. The number of cycles to fracture (NCF) was calculated by using the following formula: *NCF = time to failure (seconds) × rotational speed/60*.

### SEM analysis

Following the cyclic fatigue testing, one sample tested from each group was selected, and the fractured part of the instrument was investigated under 1000x and 2000x magnification SEM at 15 kV and 25 spot sizes (JEOL—JSM 3410, Jeol, Tokyo, Japan) to determine the pattern of fracture. The fracture surfaces of the instruments were examined for microscopic defects, such as dimples, fatigue striations, crack initiation, or circular abrasion marks (fractography), as it was mentioned in a previous study [30].

### Statistical analysis

The distribution of data was determined with histogram graphs and the Kolmogorov-Smirnov test. Descriptive analysis is shown as the means, standard deviation, and medians. The significance level was set at p<0,05.

### AFM scanning

Scanning data before and after using each instrument were subjected to a dependent sample t-test to compare the variance between groups and repetitive measuring analysis to compare the means of the test groups.

### Cyclic fatigue testing

The nonparametric values between the two groups were subjected to the Mann-Whitney U test, whereas the Kruskal-Wallis test was used for comparisons between groups more than two groups.

## Results

### AFM scanning

**Fig 2:**
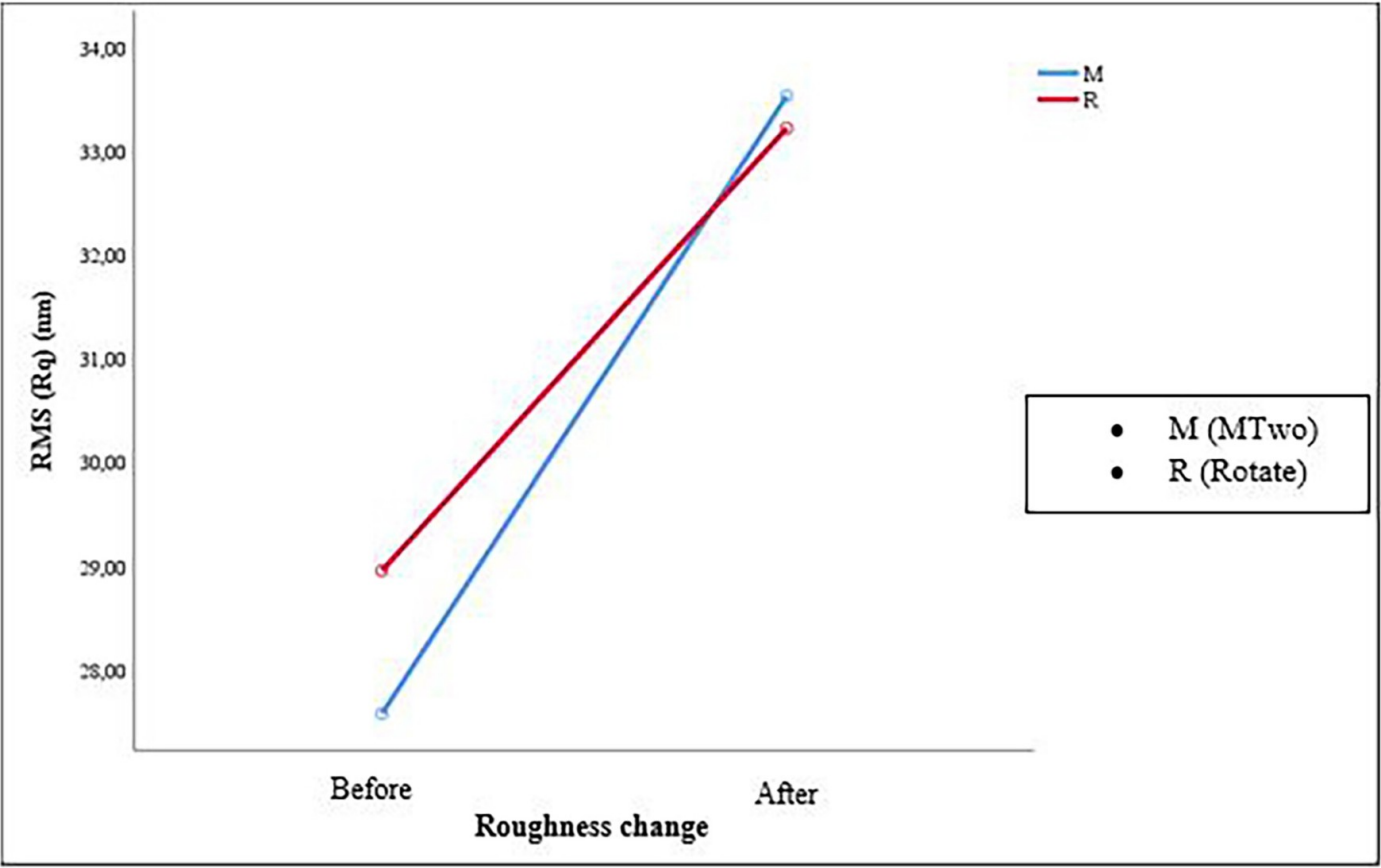
Roughness change (nm) in each tested shaping system in RMS before and after use.

The mean and median values representing the change in Ra and RMS are given in Table 1. The Ra and RMS values in each test group increased after the use of instruments compared to the unused ones (p<0,05) (Fig 2). However, no significant difference was observed between the tested groups in terms of roughness change.

**Table 1:** Roughness change in means of Ra (nm) and RMS (nm) for both tested systems. (different capital superscript letters indicate within group significancy, different lower-case superscript letters indicate significancy between groups).

		MTwo		Rotate	
		*Mean±SD*	*Median*	*Mean±SD*	*Median*
**Ra (nm)**	***1***^***st***^ ***Scan***	19,12±1,98^Bx^	19,8 (15,27–20,8)	21,24±4,53^Fx^	21,04 (15,36–26,57)
	***2***^***nd***^ ***Scan***	23,76±3,78^Ay^	24,38 (17,5–28,78)	24,17±4,68^Ey^	26,39 (16,45–27,93)
**RMS (nm)**	***1***^***st***^ ***Scan***	27,58±4,10^Dt^	26,9 (21,28–32,43)	28,96±6,96^Ht^	28,01 (19,61–36,7)
	***2***^***nd***^ ***Scan***	33,54±6,86^Cz^	33,21 (22,93–43,38)	33,23±6,94^Gz^	36,16 (20,6–39,24)

### Cyclic fatigue testing

The mean and median values presenting the change in values (NCF) in regard to the angle of curvature are shown in Table 2. For Rotate files, the NCF was significantly lower at 90° compared to 45° and 60° (p<0,001), whereas no significant difference was seen for the latter. Similarly, the NCF values for MTwo files were significantly lower at 90° than those at 45° and 60° (p<0,001), whereas no significant difference was seen between 45°and 60°.

**Table 2:** NCF values for each angle of curvature in regard to the shaping system and the angle of curvature (different capital superscript letters indicate significance p<0,05, AOC: angle of curvature).

	*Mean±SD*		*Median*		p
AOC	*MTwo*	*Rotate*	*MTwo*	*Rotate*	
45°	151,67±26,83^A^	505,42±102,94^C^	145 (110–195)	490 (325–705)	<0,001*
60°	128,25±17,67^A^	407,92±62,36^C^	122,5 (100–160)	415 (300–500)	<0,001*
90°	94,17±23,56^B^	288,00±57,29^D^	93 (64–130)	280 (205–390)	<0,001*
p	<0,001*	<0,001*	<0,001*	<0,001*	

NCF values with regard to the shaping system are also shown in Table 2. For all angles of curvature degrees, NCF was found to be significantly higher for Rotate files than for MTwo files (p<0,001).

### SEM analysis

**Fig 3:**
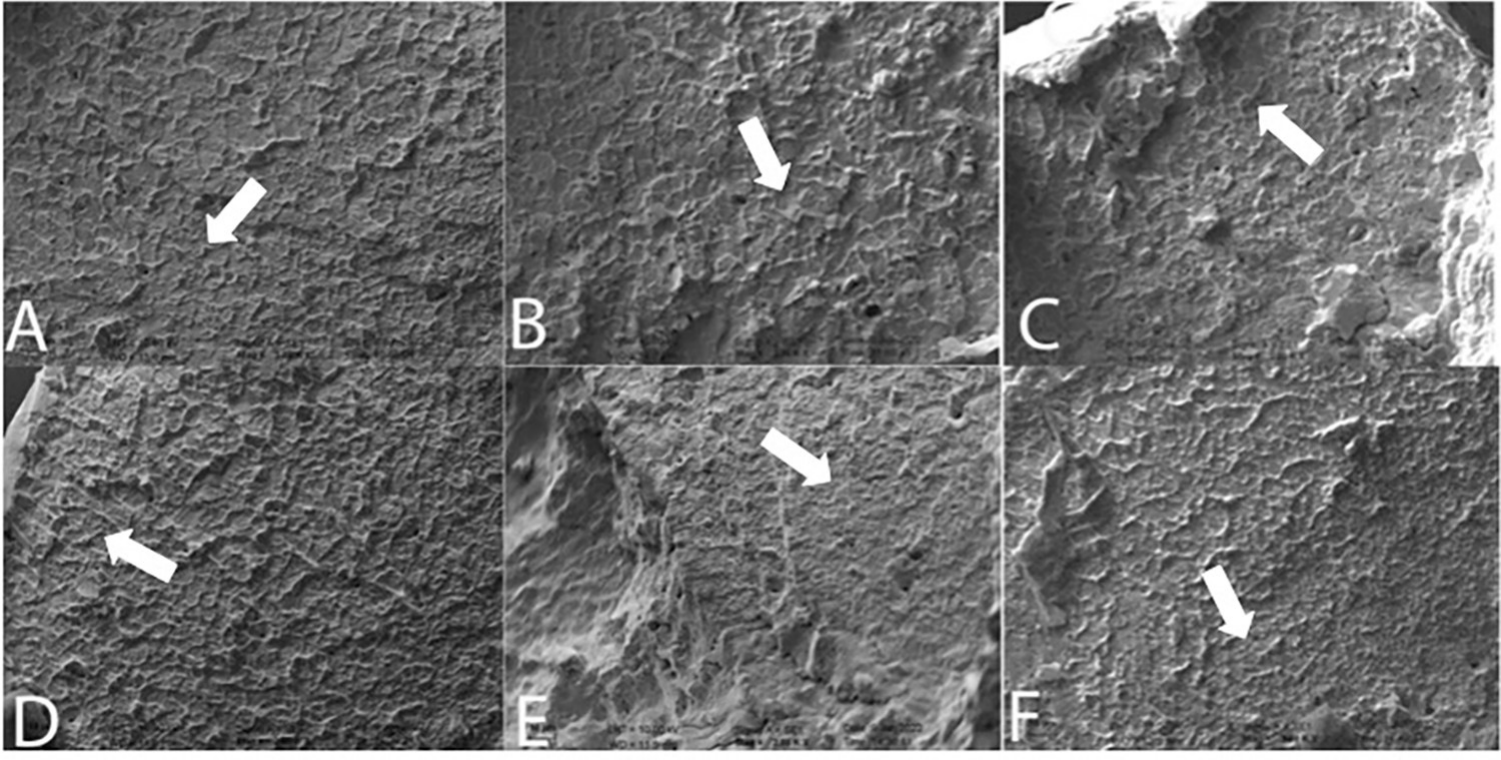
Representative SEM images for fracture planes after cyclic fatigue testing: (A-C) Upper row for MTwo at 45-60-90 degrees of curvature, respectively; (D-F) Lower row for Rotate at 45-60-90 degrees of curvature, respectively. *(2000x magnification)*.

All instruments analyzed qualitatively and they presented similar fracture planes regardless of the degree of curvature. The fractographic examination revealed fatigue failures. The fracture surfaces showed elongated circular dimples, indicating ductile fracture (Fig 3A–3D *shown with an arrow*). Additionally, the presence of circular abrasion marks indicated shear loading (Fig 3B–3E *shown with an arrow*). Fatigue striations (Fig 3C–3F *shown with an arrow*) suggested a failure due to permanent deformation.

## Discussion

Topographic irregularities on the surface of NiTi endodontic instruments as a result of machining applications, autoclaving cycles, or root canal shaping were previously reported [5, 16]. These irregularities may have a significant impact on fracture resistance as well as bacterial adhesion to the material surface [31]. Therefore, improving the surface of NiTi files through additional treatments such as polishing or coating was proposed to be beneficial [7, 8].

Recent research has shown a growing interest in using three-dimensional optical profilometry instead of AFM in roughness testing [32]. This tendency could be attributed to the profilometer providing broader scanning, whereas the AFM scanning area is much smaller. However it has been reported that AFM is a sensitive and reliable method and it could obtain qualitative and quantitative topographical data on the surface roughness of NiTi instruments [14, 33]. In our preliminary testing, we could not obtain a detailed scan of the desired area due to the twisted shape of the rotary instrument with blades and flutes. Therefore, we chose AFM to obtain three-dimensional images for both shaping systems (Fig 2).

**Fig 4:**
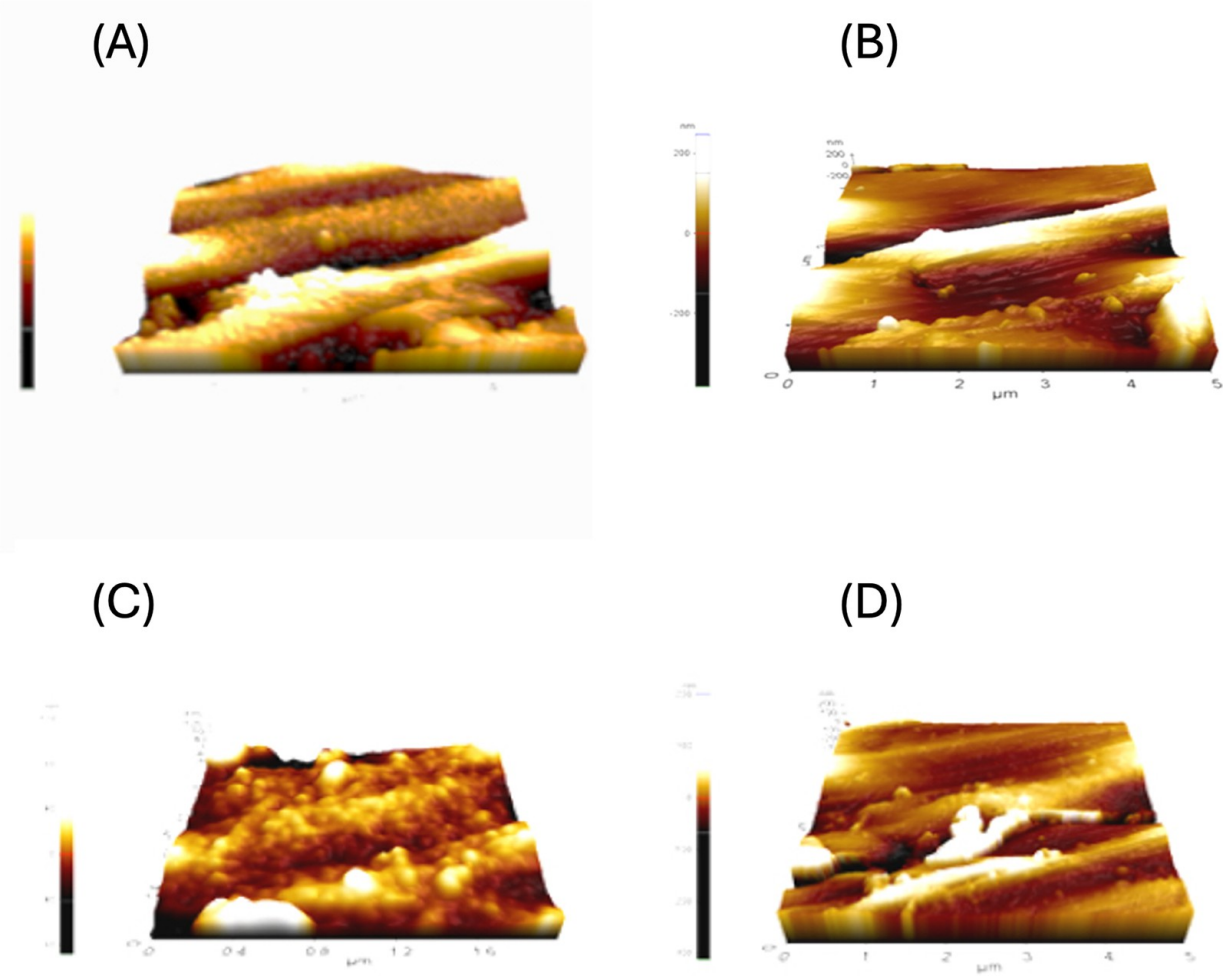
Representative images for AFM scanning (5x5nm area size): (A, B) Surfaces of Rotate and MTwo instruments before use, (C, D) surfaces of Rotate and MTwo instruments after use.

In our study, both instruments showed an increase in surface roughness after use. This finding was also supported by Yamazaki-Arasaki et al. [16], who also presented an increase in wear following the use of NiTi instruments. Although the ‘blue-treated’ files presented similar surface characteristics to their conventional counterparts [11], alterations on the surface may arise due to manufacturing processes [8]. Nevertheless, in our study, Rotate files manufactured from a heat-treated NiTi alloy presented higher Ra and RMS values, rejecting the null hypothesis, and correlated with previously reported roughness values of thermally treated instruments [34]. Additionally, the surface of the Rotate files is further coated during manufacturing, which may have increased the Ra and RMS values further compared to MTwo files. Ertugrul et al. [18] performed EDX analysis on the surface of Rotate and MTwo files and presented higher titanium levels (wt%) compared to MTwo files, indicating further surface treatment with titanium oxide coating. Our AFM scans revealed that the Rotate files consisted of protuberances (Fig 4) compared to MTwo files to support this knowledge. Nevertheless, the characterization of such surfaces with additional methods, such as XPS (X-ray photoelectron spectroscopy), may be beneficial in future studies.

Fracture in rotary instruments occur due to either torsional or flexural fatigue and may affect the treatment prognosis [34]. Cheung *et al*. [31] reported that flexural fatigue was the most frequently (98%) observed mechanism in failed instruments. Therefore, the instrument’s resistance to cyclic fatigue is important for a satisfactory treatment outcome.

To date, several devices and methods have been used to investigate the cyclic fatigue resistance of NiTi rotary endodontic instruments. In nearly all studies reported in the endodontic literature, the rotating instrument was either confined in a glass or metal tube, in a grooved block-and-rod assembly, or in a sloped metal block consisting of artificial canals [35]. These canals are expected to resemble clinical conditions as in a reliable canal curvature and trajectory with a canal space adapted to the instrument dimensions. More recently, Plotino *et al*. proposed a specifically constructed cyclic fatigue device adaptable to the dimensions of the tested instrument, where they suggested that a precise trajectory that sufficiently restricts the working portion of the instrument disables the file from regaining its original shape during testing [36]. In our study, we followed the parameters described above to construct artificial canals onto stainless steel blocks for each tested instrument size (0.06 and 0.05 tapers) as used in previous studies [18, 37]. Furthermore, Pruett *et al*. [29] described the maximum load for stress as being close to the arc midpoint of the canal. In our study, this point was 5 mm from the tip of every artificial canal in the testing device for instrument comparability.

Our results showed the highest NCF values at a lower curvature angle of 45° for both instruments, whereas severe curvature of 90° was the lowest, revealing that the severity of canal curvature had an influence on the fatigue resistance. This was similar to Pirani et al. [38] who also presented lower cyclic resistance when rotary files used in 60° compared to that of 45°. The NCF values were also higher for Rotate files than for the Mtwo files which can be attributed to the titanium-oxide on the instrument surface. Similarly, a study comparing the cyclic resistance of Rotate files to MTwo, One Curve, and TF Adaptive instruments presented higher cyclic resistance in Rotate files [18].

Examining the fracture surface at higher magnifications is necessary to determine the origins of material failure [31]. In this study, the fracture plane following cyclic fatigue testing was investigated under SEM to clarify the failure patterns. Fatigue striations were visible on the fractured planes (Figs 3C–4D), indicating failure due to fatigue. Cheung *et al*. [31] conducted a fractographic examination with SEM on fractured ProTaper instruments collected after clinical use and revealed that fatigue striations were dominantly present on the fracture planes. Another study by Alapati *et al*. [2] examined failed instruments under SEM and revealed a higher number of dimpled rupture marks, indicating failure after significant permanent deformation, which was parallel to our findings (Fig 3). However, the researchers used a higher magnification (up to x7500) to analyze the intergranular fracture even further. In our study, the highest magnification was x2000 which was to obtain an elaborate view of the fracture plane. In contrast to their results, we did not observe machining grooves on the instrument surfaces. This can be attributed to the examination plane, as we only included the horizontal view for investigation in our study, whereas machining grooves were mostly visible on the lateral view planes. No significant differences were seen on the fracture planes of the different angles of curvature. This finding could be attributed to the same permanent deformation as a result of cyclic fatigue testing, indicating that the severity of curvature may not be an influencing factor on the failure patterns of broken instruments.

The use of static cyclic fatigue testing has recently been criticized in regard to experimental conditions that are widely inconsistent. It was previously reported by Dederich&Zakariasen that an addition of axial movement to the test may result in an increase in cyclic fatigue resistance [39]. This was followed by Pruett et al., who stated that a static cyclic fatigue test cannot be seen as a fully appropriate test in investigating the dynamic properties of a NiTi engine-driven instrument [29]. However, these opinions were limited to testing mechanisms that allowed the instrument to rotate freely. A static test designed in a hollow tube aims to overcome previously addressed concerns while falling into other categories that might affect the outcome, such as the trajectory of the file, the material of the tube, radius, and angle of curvature or liquid used in lubrication [40]. In our study, we tried to minimize these variabilities by taking into account the indications given by Plotino *et al*. [35]. Nevertheless, a lack of vertical motion as a dynamic test model may have affected our results in reflecting clinical viewing [40].

Another relevant shortcoming of our study is that room temperature was used in the study design. The environmental temperature has gained critical attention over the years, as the instrument presents different behavior in room or body temperature [41]. Grande *et al*. presented higher cyclic fatigue resistance for both traditional and thermally treated instruments tested at lower temperatures [42]. The lower temperature exhibits more martensitic features that grant the instrument a longer lifespan; however, in our study, we aimed to evaluate the effectiveness of the ‘blue treatment’ on instruments’ cyclic fatigue resistance when tested in various curvatures and not prioritize the ‘temperature’ effect.

Manufacturers of endodontic rotary files introduce shaping systems that differ in taper, size, or cross-sectional design with the aim of feasibility, efficiency, and usability for clinicians. Therefore, an in vitro study design may include a comparison of rotary files even though they differ in structural design [27, 32]. In this study, MTwo system was compared with Rotate files mainly because it was considered that the Rotate system was an improvement of the MTwo files. Both systems share similarities, such as the file sequence or cross-sectional design, as well as differences, such as NiTi alloy or taper size [43]. Similar to our study, De-Deus et al. compared Reciproc and Reciproc Blue files, in which the latter was an improvement to the former with some structural differences [11].

In our study, due to difficulties in maintaining the AFM probe in position, a #20 file from both shaping systems was selected rather than a larger instrument. A larger instrument would involve a stiffer material with more friction, resulting in higher roughness values or cyclic fatigue resistance. However, we think the processes of manufacturing each system have more impact on our results than the tip size; hence, the tip size is negligible. The scanning area was also designated as 5 mm above the apex of the instrument since it was not possible to keep the instrument in position at lower areas [16]. However, this situation did not interfere with the results mostly because regarding our cyclic fatigue testing, the center of curvature was placed 5 mm above the apex. Another limitation was in creating optimal environmental conditions.

The possible clinical impact of a surface coating such as the ‘blue treatment’ should be evaluated in future studies with different aspects. The present study provided encouraging results that the treatment lowers instrument roughness with an increase in cyclic fatigue resistance that can motivate clinicians to use Rotate instruments during root canal shaping.

## Conclusions

The roughness increased in used instruments compared to unused ones, whereas no significant difference was seen between the two shaping systems. Rotate files presented a statistically better cyclic fatigue resistance compared to that of MTwo, and the resistance decreased as the severity of the curvature increased for both groups. The fracture plane was not influenced by the severity of curvature and both instruments presented similar features of failure patterns.
